# Reduced left ventricular dimension and function following early life stress: A thrifty phenotype hypothesis engendering risk for mood and anxiety disorders

**DOI:** 10.1016/j.ynstr.2017.01.001

**Published:** 2017-01-03

**Authors:** Jeremy D. Coplan, Anna V. Rozenboym, Sasha L. Fulton, Venkatesh Panthangi, Jean Tang, Lakshmi Thiramangalakdi, Tarique D. Perera, Yang Liu, Haroon Kamran, Michael J. Owens, Charles B. Nemeroff, Leonard A. Rosenblum, John G. Kral, Louis Salciccioli, Jason Lazar

**Affiliations:** a Department of Psychiatry and Behavioral Sciences, State University of New York (SUNY) -Downstate, Brooklyn, NY, United States; b Kinsborough Community College, Brooklyn, NY, United States; c Department of Psychiatry, New York State Psychiatric Institute, New York, NY, United States; d Division of Cardiology, Department of Medicine, SUNY-Downstate, Brooklyn, NY, United States; e Department of Psychiatry and Behavioral Sciences, Emory University School of Medicine, Emory, GA, United States; f Department of Psychiatry and Behavioral Sciences, University of Miami Health Systems, Miami, NY, United States; g Departments of Internal Medicine and Surgery, SUNY-Downstate, Brooklyn, NY, United States

## Abstract

**Background:**

Early life stress (ELS) in macaques in the form of insecure maternal attachment putatively induces epigenetic adaptations resulting in a “thrifty phenotype” throughout the life cycle. For instance, ELS induces persistent increases in insulin resistance, hippocampal and corpus callosum atrophy and reduced “behavioral plasticity”, which, taken together, engenders an increased risk for mood and anxiety disorders in humans but also a putative sparing of calories. Herein, we test the hypothesis whether a thrifty phenotype induced by ELS is peripherally evident as hypotrophy of cardiac structure and function, raising the possibility that certain mood disorders may represent maladaptive physiological and central thrift adaptations.

**Methods:**

14 adult bonnet macaques (6 males) exposed to the maternal variable foraging demand (VFD) model of ELS were compared to 20 non-VFD adult subjects (6 males). Left ventricle end-diastolic dimension (LVEDD), Left ventricle end-systolic dimension (LVESD) and stroke volume (SV) were calculated using echocardiography. Blood pressure and heart rate were measured only in females. Previously obtained neurobehavioral correlates available only in males were analyzed in the context of cardiac parameters.

**Results:**

Reduced LVESD (p < 0.05) was observed when controlled for age, sex, body weight and crown-rump length whereas ejection fraction (EF) (p = 0.037) was greater in VFD-reared versus non-VFD subjects. Pulse pressure was lower in VFD versus non-VFD females (p < 0.05). Male timidity in response to a human intruder was associated with reduced LVEDD (p < 0.05).

**Conclusions:**

ELS is associated with both structural and functional reductions of left ventricular measures, potentially implying a body-wide thrifty phenotype. Parallel “thrift” adaptations may occur in key brain areas following ELS and may play an unexplored role in mood and anxiety disorder susceptibility.

## 1 Introduction

Early-life exposure to stress is a well-known risk factor for psychiatric disorders later in life, including mood and anxiety disorders (Heim and Nemeroff, 2001). Exposure to stress during childhood has also been linked to a greater prevalence of cardiovascular risk factors, premature coronary artery disease and an increased risk for cardiovascular events in adulthood (Felitti et al., 1998). Specifically, preclinical (Loria et al., 2013) and human studies have shown higher rates of obesity (Alciati et al., 2013), smoking (Anda et al., 1999), diabetes (Kaufman et al., 2007), hypertension (Loria et al., 2010, Alastalo et al., 2013, Nuyt, 2008) and myocardial infarction (Dong et al., 2004) many years following exposure to early life stressors (Steptoe and Kivimäki, 2012). Stressors include separation from parents, parental death and other adverse experiences (Bercovich et al., 2014). While early adversity is a well-known cause of cardiomyopathy (Steptoe and Kivimäki, 2013), few studies have assessed the long term effects of ELS directly on cardiac structure and function (Carlier et al., 1988). Underdevelopment of cardiac structure and/or function may represent “thrift” oriented developmental programming, raising the possibility that neurotrophic compromise may be implicated as a strategy for saving energy.

Among the first clinical syndromes linking early life stress (ELS) to blunted somatic growth was psychosocial dwarfism (Money, 1977), a clinical entity observed in childhood caused by profound emotional deprivation or stress with an associated growth hormone deficiency (Albanese et al., 1994). Reversal of stunted growth occurred only with emotional normalization and not with nutritional provisions alone (Reinhart and Drash, 1969). Epigenetic studies in maltreated children examining genome-wide methylation differences (Weder et al., 2014) identified three genes relevant to the stress response, neural plasticity, and neural circuitry. In maltreated children, methylation of 32 of an identified 150 CpG sites located in these three genes specifically related to cardiac development (Weder et al., 2014).

The persistent disruption of central (Coplan et al., 1996) and peripheral (Kaufman et al., 2007) biological measures observed to date using our nonhuman primate model of ELS are accompanied by epigenetic effects at the serotonin transporter gene promoter region as well as genome-wide methylation effects (Kinnally et al., 2011).

We report on adult LV structure and function in an animal model of ELS, specifically bonnet macaques exposed to variable foraging demand (VFD), a paradigm in which infants are reared by mothers subjected to an experimentally-induced perception of food uncertainty without caloric deprivation (Rosenblum and Paully, 1984). VFD-reared subjects exhibit timidity (Jackowski et al., 2011) and loss of “behavioral plasticity” -- a term applied to adaptive modifications of behavior (Molteni et al., 2004) -- in response to a human intruder (Rosenblum et al., 2001). Moreover, VFD-reared macaques exhibit persistent elevations in cerebrospinal fluid (CSF) concentrations of corticotropin releasing-factor (CRF) (Coplan et al., 2005), high levels of which were found to predict components of the metabolic syndrome (Perera et al., 2011). Reductions in corpus callosum cross-sectional area are also observed (Jackowski et al., 2011) -- the latter finding has been replicated in rhesus macaques (Sánchez et al., 1998) and young humans (Teicher et al., 2004) exposed to ELS. Should the latter parameters – “behavioral timidity”, increased CSF CRF concentrations and reduced corpus callosum cross sectional area – be significantly related to cardiac parameters reflecting decreased dimension and function, preliminary support would be provided for the view that neurotrophic compromise may represent one consequence of thrift following early life stress.

Investigators have utilized nonhuman primates as models for human mood and anxiety disorders (Coplan et al., 2014). However, macaques are also remarkably similar anatomically to humans with regard to cardiovascular physiology and metabolism (Lazar et al., 2008, Haider et al., 1977, Vaitkevicius et al., 2001, Kaufman et al., 2005). Our group has used echocardiography to characterize left ventricular size and function in a laboratory colony of adult bonnet macaques and has reported reference values for LV systolic and diastolic dimension across the life span (Lazar et al., 2008). Furthermore, the very low incidence of atherosclerosis in chow-fed bonnet macaques provides an opportunity to study the relation between early life stress and cardiac structure and function in a model impervious to ischemic heart disease (Lazar et al., 2009).

Although infants reared by mothers exposed to VFD do not experience any lag in normative weight gain (Coplan et al., 1996), several lines of evidence, outlined below, suggest that body-wide adaptations shift the bonnet macaque's physiology towards an energetically “thrifty” mode across the life cycle. Hales and Barker first termed the “Thrifty Phenotype Hypothesis” to explain the origins of Type II Diabetes Mellitus as the consequence of long-term adaptations to early life caloric deprivation (Hales and Barker, 1992). However, recently the concept has been theorized as a programmatic reconfiguration following ELS leading to a ‘”Thrifty Psychiatric Phenotype” in which the convergence of caloric deprivation and early life emotional deprivation may engender an uncertainty of caloric access (Garcia-Rizo et al., 2015, Ockenburg et al., 2015). This view is supported by several lines of evidence in our early life stress model, including 1) persistent hypocortisolemia (Coplan et al., 1996) leading to reduced tissue catabolism for gluconeogenesis, 2) elevated concentrations of CSF CRF (Coplan et al., 1996), a stress neuropeptide with anorexogenic effects (Pelleymounter et al., 2000), 3) CRF inversely predicting the trophic signaling of GH in response to the GH secretagogue, clonidine (Coplan et al., 2000) and 4) insulin resistance and features of the metabolic syndrome (Kaufman et al., 2007). Based on these considerations and other putatively thrift-driven adaptations observed in VFD subjects such as decreased hippocampal volume (22), we hypothesized that LV cardiac dimension may be persistently “hypotrophic”. Certainly, longstanding reductions in LV capacity would be consistent with a calorically thrifty adaptation. Our primary hypothesis was that VFD-rearing would lead to a persistent reduction in LV dimension, which would necessitate an increase either in ejection fraction (EF) or heart rate to maintain adequate stroke volume. In addition, evidence of a relationship between left ventricular dimension/function and previously obtained biobehavioral markers of affective dysregulation would support the hypothesis of wide-spread thrift adaptations following ELS. We therefore explored whether alterations in LV dimension/function would predict greater timidity in response to a human intruder, relative elevations of CSF CRF concentrations and relative reductions in corpus callosum white matter cross-sectional area.

## 2 Methods

### 2.1 Colony

Characteristics of the State University of New York Downstate Medical Center primate colony have been described previously (Kaufman et al., 2005). The colony consisted of laboratory-born and raised bonnet macaques (*Macaca radiata*) living either in social groups of 6–10 (females) or singly-housed adult males in full view of their peers maintained on standard commercial monkey chow who had been reared under social conditions until fully mature at which point injurious fights may occur. All procedures were performed in careful accordance with the Guide for the Care and Use of Laboratory Animals (https://www.aaalac.org/resources/theguide.cfm). The State University of New York Downstate Medical Center Institutional Animal Care and Use Committee (IACUC) approved the study.

Subjects: 34 adult bonnet macaques (22 female, 12 male) of which 14 (6 males) were VFD-reared and 20 (6 males) not exposed VFD served as subjects. Table 1 provides means (SD) of age, weight and crown-rump length for each of four groups (VFD/non-VFD-- male/female).

**Table 1:** Means and standard deviations of age, weight and crown-rump length of differentially-reared nonhuman primate groups by sex.

Group	Mean age (years) a	Mean weight (kg) b	Mean crown rump length (cm)^c^	N
Male non-VFD	9.47 ± 2.31	9.22 ± 2.71	50.57 ± 2.27	6
Male VFD	7.95 ± 0.50	8.47 ± 1.87	50.04 ± 1.91	6
Female non-VFD	4.9 ± 2.8	5.91 ± 2.19	43.52 ± 1.94	14
Female VFD	4.5 ± 1.4	6.80 ± 1.53	45.38 ± 1.68	8
All Groups	6.15 ± 2.88	7.15 ± 2.41	46.31 ± 3.53	34

± = standard deviation; VFD = variable foraging demand reared.

a Sex Effect -- F_(1,30)_ = 25.11; p < 0.001 M > F. No rearing or rearing x sex effects.

b Sex Effect -- F_(1,30)_ = 11.00; p < 0.001 M > F. No rearing or rearing x sex effects.

c Sex Effect -- F_(1,30)_ = 72.18; p < 0.001 No rearing or rearing x sex effects.

### 2.2 Rearing methods

For VFD rearing exposure (Rosenblum and Paully, 1984), mothers were confronted with an environment in which adequate amounts of food were always available but in which the amount of time and effort necessary to obtain daily rations were unpredictable. The VFD maternal food procurement schedule consisted of alternating blocks of 2-weeks in which food was easy to find (Low Foraging Demand; LFD), and 2-weeks in which food procurement was difficult, involving more time and effort (High Foraging Demand; HFD). Beginning with LFD, a total of four, 2-week periods of LFD and four, alternating 2-week periods of HFD comprised the 16 week VFD experimental period. The age of the infants at the time of onset of the VFD procedure is ∼3 months of age.

To vary foraging demand, a simple device, referred to as a “foraging cart” was implemented in such a way that food could either be buried in wood chips (HFD) or left freely exposed in containers within (LFD). Animals were required to manually search for and retrieve the apportioned food through multiple apertures on both sides of the cart. Further details of the “Foraging Cart” and the VFD procedure may be found in Andrews and Rosenblum (1991).

### 2.3 Morphometry, blood pressure and heart rate

Anesthesia was induced by intramuscular ketamine (15 mg/kg) and repeated in small amounts as clinically indicated throughout the procedure. Immediately after sedation, each monkey was weighed; crown rump length was measured; and heart rate (HR), systolic blood pressure (BP), and diastolic BP were recorded by sphygmomanometry of the right lower extremity (only available in females). Pulse pressure was determined as: systolic minus diastolic BP. Heart rate was determined through echocardiographic measures.

### 2.4 Echocardiography

Echocardiography (Model Sonos 5500 machine with a 3.5- to 5.5-MHz transducer, Phillips, Andover, MA) was performed in all 34 monkeys by an experienced echocardiographer blind to rearing condition. Each study was inspected carefully to assure optimal imaging and was recorded on high-definition video tapes. Standard echocardiographic images were obtained. Left ventricular dimensions were measured from M-mode and 2-dimensional parasternal long-axis and apical 4-chamber axis images according to the American Society of Echocardiography standards (Lazar et al., 2008). Three measurements were made, and average values were recorded. LV mass and EF were calculated by the American Society of Echocardiography-corrected cube formula and adapted to old world monkeys (Lazar et al., 2008, Lang et al., 2005). LV mass indexed by body surface area, LV fractional shortening, septal wall thickness, posterior wall thickness, LV end-diastolic dimension (LVEDD), and LV end-systolic dimension (LVESD) were determined for each monkey.

### 2.5 Neurobehavioral measures

Methods and results have been reported in detail: Behavioral response to a human intruder [mean (SD) age = 8.7 (1.7) years; N = 12], MRI [mean (SD) age = 4.4 (1.9) years; N = 12] for cross-sectional area of corpus callosum (Jackowski et al., 2011) and CSF CRF concentrations [mean (SD) age = 3.2 (0.6) years; N = 11] (Coplan et al., 2011) were available for analysis.

### 2.6 Statistical analyses

Variables were tested for homogeneity of variance and inspected for outliers. A general linear model (GLM) employed a factorial design examining for rearing effects, sex effects and their interaction while using age, weight, and crown rump length as covariates. The adaptation of human echocardiography to the bonnet macaque, with its species-specific morphometrics, was reported in Lazar et al. (2008). In that colony-wide study, it was determined that body surface area, except for LV mass, was not as predictive of LV dimension as age, body mass and crown-rump length (Lazar et al., 2008). The latter three variables were used as covariates and were based on prior echocardiographic study of this population (Lazar et al., 2008). Left ventricle end-diastolic dimension (LVEDD) and left ventricle end-systolic dimension (LVESD) were used as repeated measures in the GLM. To validate the rationale of the repeated measures, we correlated LVESD with LVEDD and confirmed that the variables in question were dependent [Pearson's r = 0.77; N = 34; p = 0.001]. LV ejection fraction (EF) was analyzed similarly but used as a single dependent variable. Stroke volume (SV) was determined by LVEDD – LVESD and treated as a dependent variable. Systolic and diastolic BP were used as repeated measures, whereas HR and pulse pressure were analyzed as single dependent variables although, since these measures were only available in females, sex was not included in the GLM as a categorical variable. Total arterial compliance was determined in females by dividing SV by pulse pressure and also treated as a dependent variable. The same GLM method was applied for the remaining echocardiographic variables. Pearson correlations were performed in males between LV dimension/function parameters – LVEDD, LVESD, SV and EF -- and CSF CRF and corpus callosum cross-sectional, rendering a total of 8 correlations. Because response to a human intruder had previously been dichotomized into timid versus confrontational responses (Jackowski et al., 2011), t-tests compared the two behavioral responses as an independent variable and the four identified echocardiographic variables as dependent variables –LVEDD, LVEDD, SV and EF. All analyses were performed using Statistica version 12. A two-tailed p value of p ≤ 0.05 was considered to be statistically significant. Because of the exploratory nature of the study, correction for multiple comparisons was not performed. However, to validate correlational findings, significant findings were entered into a GLM using rearing method as a control variable and effect sizes were determined (partial η^2^).

## 3 Results

### 3.1 Echocardiographic measures in males and females

#### 3.1.1 Age, sex and morphometric data

All variables demonstrated normality of distribution and outliers were not noted for the primary variables of interest. In Table 1, means and standard deviations of age, weight and crown-rump length are provided for the sample, divided according to sex and rearing group – VFD males, VFD females, non-VFD males and non-VFD females. GLM revealed that males were older and weighed more than females but no rearing effect or rearing x sex effects were noted for age and weight. Crown-rump length showed a sex effect (M > F) but did not exhibit rearing or rearing x sex effects. All three variables were used as covariates in all subsequent GLMs. There was no significant difference in terms of sex distribution when comparing the two rearing groups [χ 2 (df = 1) = 0.60; p = 0.44].

#### 3.1.2 Left ventricular systolic and diastolic dimension

**Fig. 1:**
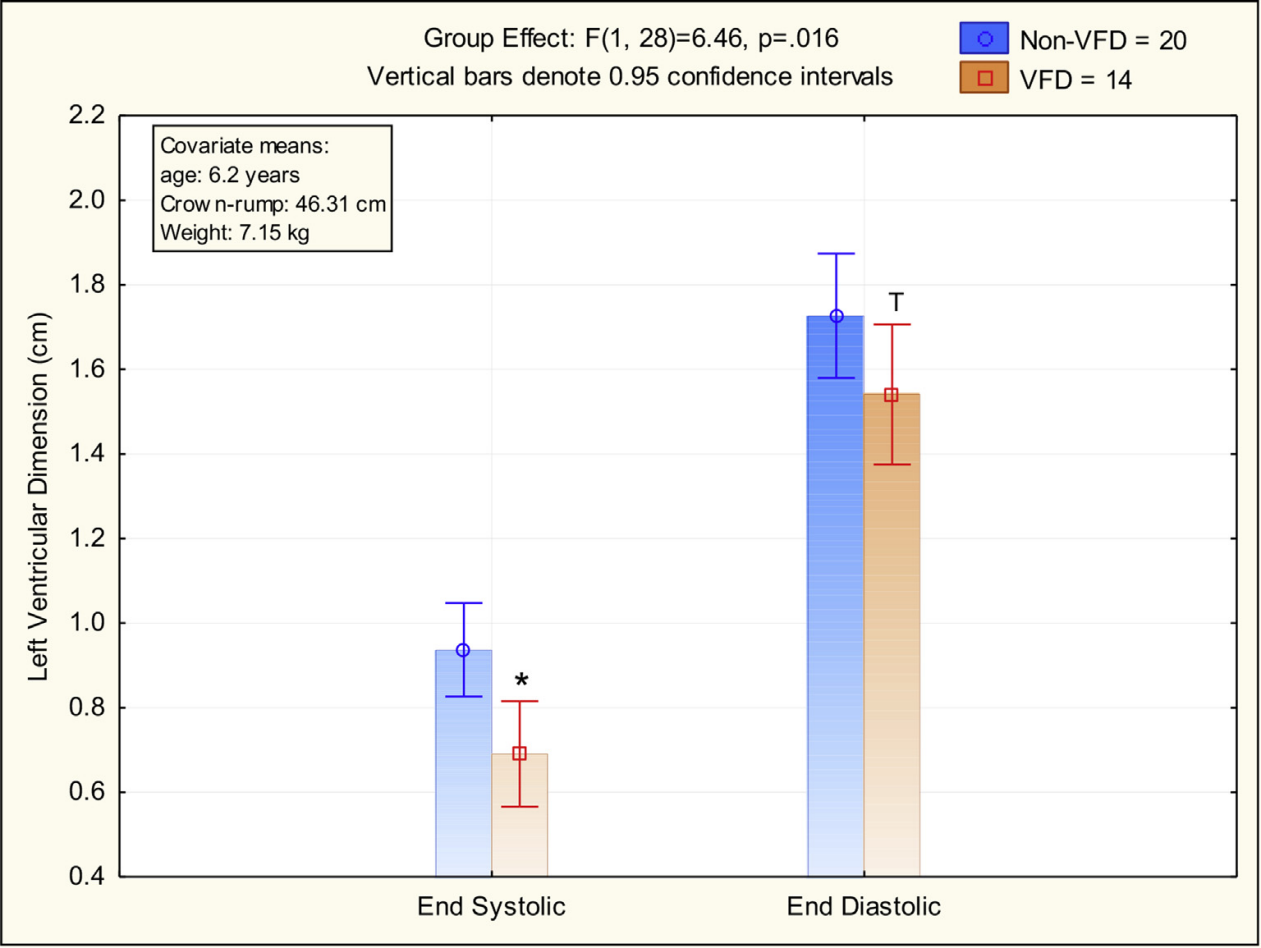
Comparison of Left Ventricular End-Diastolic (LVEDD) and End-Systolic Dimension (LVESD) Following Early Life Stress [Variable Foraging Demand-Rearing (VFD)]. Using a general linear model, adjusting for sex, age, weight and crown-rump length, an overall rearing effect was noted [F_(1,28)_ = 6.46; p = 0.02] indicating a lower overall dimension in the VFD-reared versus the normally-reared group. For univariate results, significant decrements [F_(1,28)_ = 8.93, p = 0.006] in VFD for LVESD versus non-VFD were observed but were not significant (trend)for LVEDD [F_(1,28)_ = 2.89, p = 0.10].

Using the GLM, an overall rearing effect was noted indicating overall decreases in LV dimension in the VFD-reared versus the normally-reared group (see Fig. 1). For univariate results, significant decreases [F (Heim and Nemeroff, 2001, Teicher et al., 2004) = 8.93, p = 0.006] in VFD [mean (SE) = 0.74 (0.06) cm; N = 12] versus non-VFD [mean (SE) = 0.92 (0.05) cm; N = 22] were observed for LVESD but were not significant for LVEDD [F_(1,28)_ = 2.89, p = 0.10; VFD subjects [mean (SE) = 1.57 (0.08) cm; n = 12] versus non-VFD subjects [mean (SE) = 1.76 (0.07) cm; N = 22] (Fig. 1). An overall sex effect [F_(1,28)_ = 13.07, p = 0.001] reflected sexual dimorphism, adjusted for weight and crown-rump length, with larger LV dimension in males [adjusted mean (SE) = 1.5 (0.06)] than females [adjusted mean (SE) = 1.01 (0.05) cm = ]. Weight, adjusted for sex and crown-rump length, was a significant covariate [F_(1,28)_ = 5.30, p = 0.03] reflecting larger LVEDD (r = 0.57; N = 34; p < 0.001) and LVESD (r = 0.8; n = 34; p = 0.004) in heavier animals. Because there was a concern that the age of the males (∼8.7 years) versus females (∼4.7 years) was substantial, and that sex and age were therefore confounded, we sought to examine the relative contributions to LVESD and LVEDD by sex, age and weight. Although sex [F_(1,30)_ = 7.67, p = 0.01] and weight [F_(1,30)_ = 6.02, p = 0.02] were significant predictors of both LVEDD and LVESD, age was not a significant independent predictor [F_(1,30)_ = 0.39, p = 0.54].

#### 3.1.3 Left ventricular ejection fraction

A rearing effect was noted for LVEF [F_(1,28)_ = 4.79, p = 0.037] indicating that VFD subjects [mean (SE) = 77.66 (2.66)%; N = 14] exhibited increases in comparison to non-VFD subjects [mean (SE) = 74.18 (2.96) %; N = 20]. A significant sex effect [F_(1,28)_ = 6.52, p = 0.016] was noted with females exhibiting greater % values for LVEF than males. Both age; [↑age α ↑ EF; F_(1,28)_ = 5.23, p = 0.029] and crown-rump length (↑crown rump α ↓ EF; [F_(1,28)_ = 4.43, p = 0.044]) were significant covariates.

#### 3.1.4 Remaining variables

There was no measurable rearing effect for stroke volume [F_(1,28)_ = 2.10, p = 0.15] or effects for LV mass indexed by body surface area [F_(1,28)_ = 0.88, p = 0.35], LV fractional shortening [F_(1,28)_ = 3.08, p = 0.09], right wall thickness [F_(1,28)_ = 0.76, p = 0.38] septal wall thickness [F_(1,28)_ = 0.08, p = 0.77] or posterior wall thickness [F_(1,28)_ = 0.00, p = 0.96] (means and standard errors are available on request).

### 3.2 Females – blood pressure measures and heart rate

In females, no significant grouping effects were noted for either HR [VFD mean (SE) = 198.88 (6.62); N = 8) beats/minute in comparison to non-VFD subjects (mean (SE) = 191.93 (5.01); N = 14; F_(1, 17)_ = 0.48; p = 0.49] or systolic [VFD mean (SE) = 119.88 (4.18); N = 8) mm/Hg in comparison to non-VFD subjects (mean (SE) = 127.43 (3.16); N = 14; F_(1, 17)_ = 1.36; p = 0.25] or diastolic BP [VFD mean (SE) = 71.00 (4.59); N = 8) mm/Hg in comparison to non-VFD subjects (mean (SE) = 70.43 (3.47); N = 14; F_(1, 17)_ = 0.87; p = 0.36]. There were differing relations between the rearing groups between pulse pressure and crown-rump length with a direct correlation observed in the VFD group (r = 0.8; N = 8; p = 0.02) and a non-significant inverse relation in the non-VFD group (r = −0.3; N = 14; p = 0.30). A rearing group x crown rump length interaction was noted for pulse pressure [F_(1,18)_ = 13.14, p = 0.002]. For the determination of group effects for pulse pressure, the interactive term group x crown rump length was therefore entered as a covariate. Mean pulse pressure was significantly decreased [F_(1,18)_ = 9.02, p = 0.008] in the VFD [mean (SE) = 48.87 (3.65) mm/Hg; N = 8] in comparison to the non-VFD reared group [mean (SE) = 57.00 (2.76) mm/Hg; N = 14]. To identify a potential explanation of the pulse pressure differences, a GLM was performed using systolic and diastolic BP as the repeated-measures variable, which revealed a repeated measure x rearing group effect [F_(1,18)_ = 9.02, p = 0.008], produced by a numerically greater systolic and numerically lower diastolic blood pressure in the non-VFD versus VFD females. Pulse pressure directly correlated with LVESD (r = 0.57; N = 22; p = 0.005), LVEDD (r = 0.57; N = 22; p = 0.005), SV (r = 0.52; N = 22; p = 0.01) but not LV EF (r = −0.17; N = 22; p = 0.43). We then calculated total arterial compliance (SV/pulse pressure) and found it to be similar between the two groups [F_(1,18)_ = 0.09, p = 0.76].

### 3.3 Relationship of cardiac parameters to central biobehavioral measures in males

**Fig. 2:**
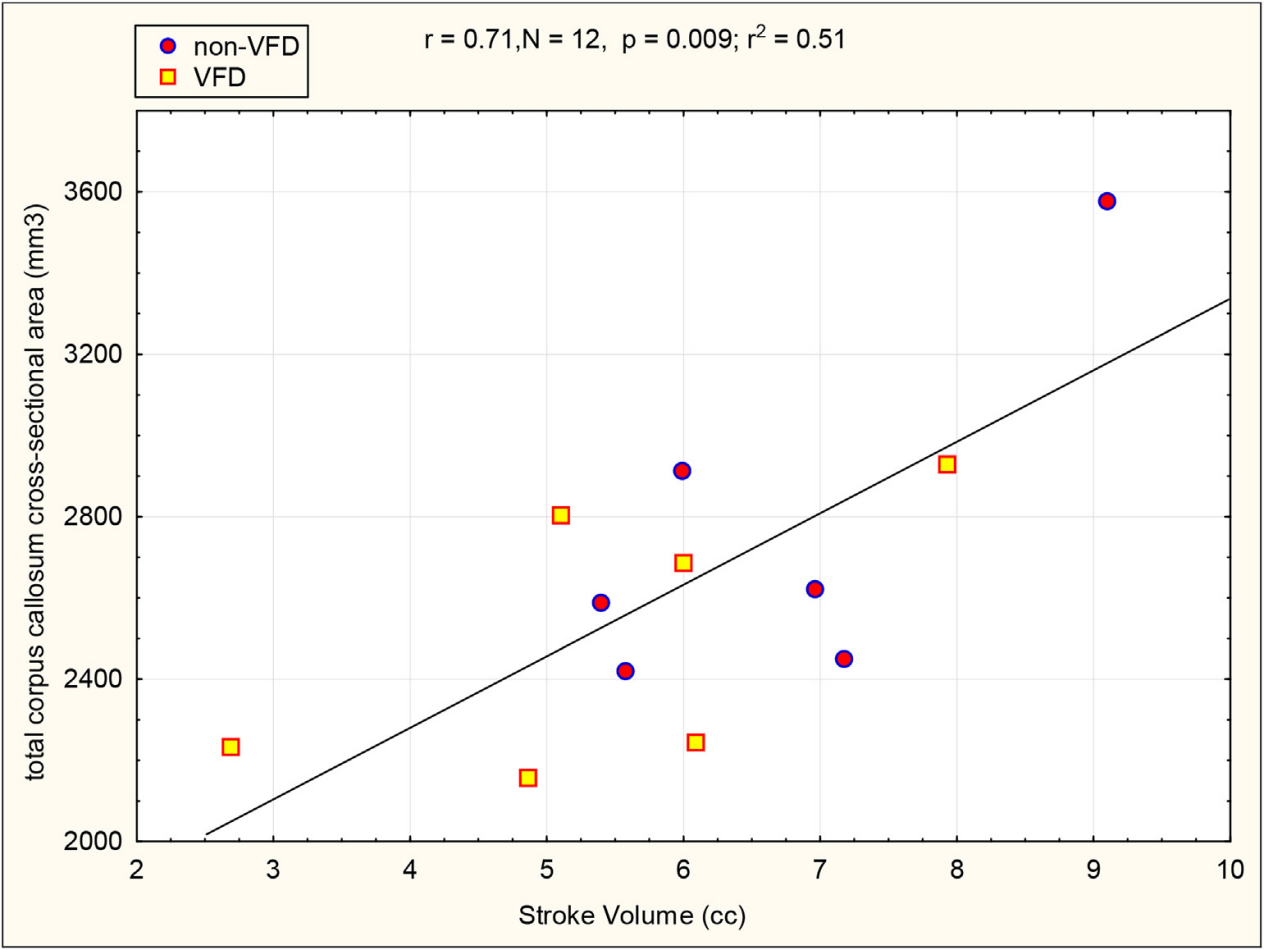
Relationship of Stoke Volume (SV) to Corpus Callosum Cross-Sectional Area in Differentially-reared Male Macaques. SV correlated positively with corpus callosum cross-sectional area (r = 0.71, N = 12, p = 0.009) without rearing effects in males. VFD = variable foraging demand rearing.

**Fig. 3:**
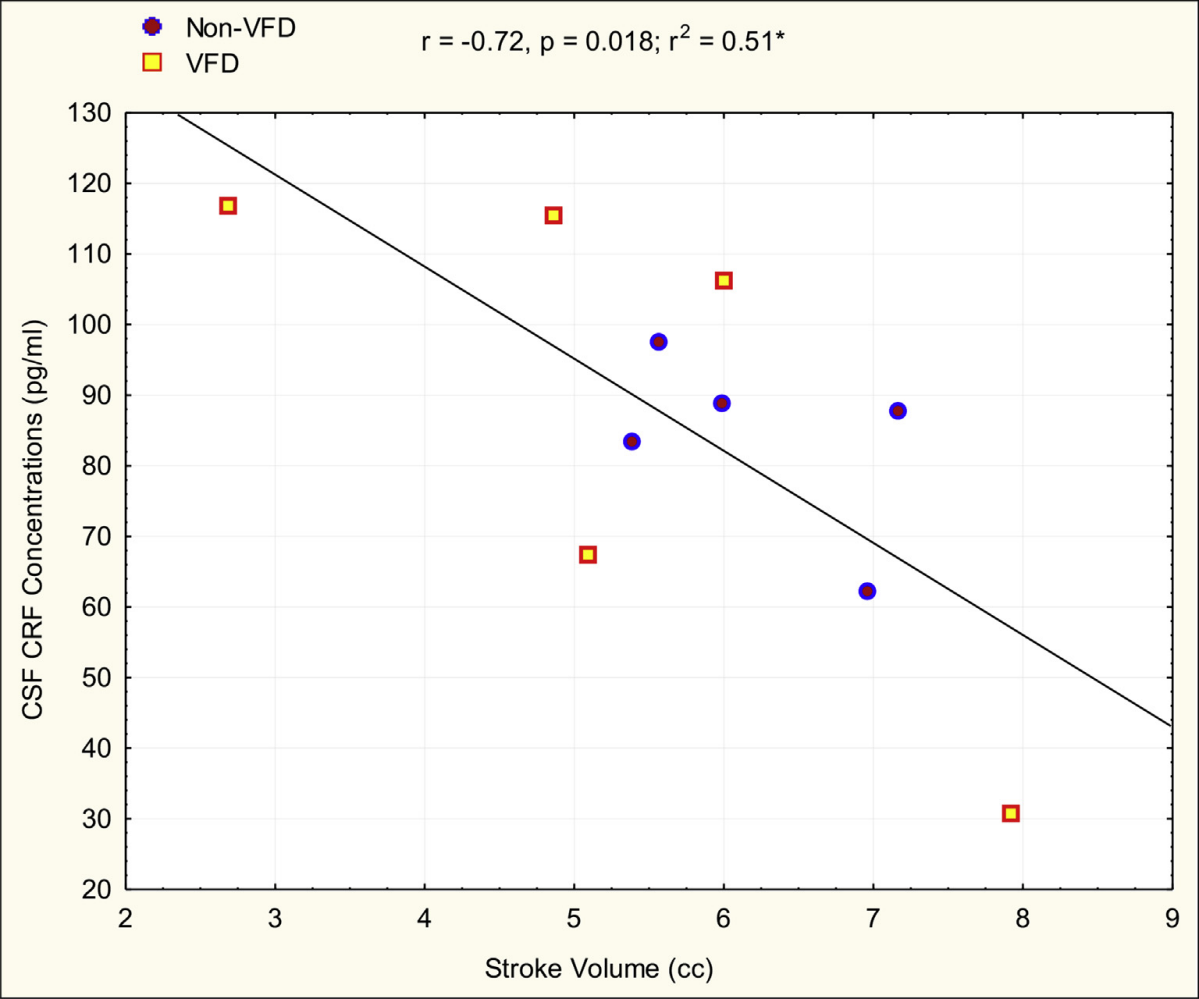
Relationship between Stroke Volume (SV) and CSF CRF (corticotropin releasing-factor) concentrations in Differentially-reared Male Macaques. There was an inverse correlation between SV and CSF CRF concentrations (r = - 0.72; N = 10; p = 0.018) [following exclusion of an outlier with 266 pg/ml of CSF CRF concentration (6.8 SD greater than the group mean)]. *- one CSF CRF concentrations outlier > 6 standard deviations from overall mean was excluded. One CSF CRF value was missing.

It should be noted that the 12 males examined in Table 2 for the relationship between cardiac parameters and biobehavioral measures, are the 12 identical males included in the VFD/non-VFD echocardiographic comparisons described above and demonstrated greater timidity in response to an intruder in VFD versus non-VFD subjects (Jackowski et al., 2011). In males (see Table 2), “timid” subjects exhibited decreases in LVEDD and SV in comparison to confrontational subjects. By contrast, differences were not noted for LVESD or EF. SV correlated positively with corpus callosum cross-sectional area (see Fig. 2, r = 0.71, N = 12, p = 0.009). There was an inverse correlation between SV and CSF CRF concentrations (r = - 0.72; N = 10; p = 0.018) [following exclusion of an outlier with 266 pg/ml of CSF CRF concentration (6.8 SD greater than the group mean)]. An inverse correlation was also noted between LVEDD and CSF CRF concentrations (r = −0.65, N = 10, p = 0.42) (one CSF CRF value excluded). Thus, three of eight neurobiological correlations were found to be significant, well in excess of the one in 20 predicted by chance. To bolster the findings, we analyzed the effect size of the aforementioned relationships described using a GLM with rearing status as a control variable. SV predicted corpus callosum cross-sectional area when controlling for rearing status [F_(1,9)_ = 7.52; p = 0.02, partial η^2^ = 0.45]. The effect size for the relationship exceeds a “large” effect size (0.14) by a factor of three (Sawilowsky et al., 2011). The rearing effect was not significant (p > 0.8). Performing a GLM on SV as an inverse predictor for CSF CRF concentrations using rearing group as a control variable revealed a larger effect size, almost greater than a “large” effect size by a factor of four [F_(1,7)_ = 8.52; p = 0.02, partial η^2^ = 0.54] whereas the rearing effect was not significant (p > 0.5) (see Fig. 3).

**Table 2:** Comparison of “confront” versus “timid” behavior in male macaques in response to a human intruder for left ventricular cardiac parameters.

	Confront N = 5	Timid N = 6	t-value	df	p
LVEDD	2.10 ± 0.22	1.84 ± 0.14	2.35	9	0.04
LVESD	1.18 ± 0.42	1.03 ± 0.21	0.80	9	0.44
SV	7.25 ± 1.46	5.10 ± 1.26	2.62	9	0.03
EF	67.74 ± 17.23	67.45 ± 12.63	0.03	9	0.97

LVEDD = left ventricular end-diastolic dimension, LVESD = left ventricular end-systolic dimension, SV = stroke volume, EF = ejection fraction.

## 4 Discussion

The current study demonstrates that early life stress in bonnet macaques in the form of VFD-rearing produced persistent LV hypotrophy as evidenced by a significant decrease in LVESD but not LVEDD. LV ejection fraction (EF) in VFD-reared subjects was increased in comparison to non-VFD reared monkeys. Despite greater LV EF in VFD subjects, LV stroke volume (SV) was equivalent in the two groups, suggesting that reduced LV cardiac dimension required a greater LV EF to maintain equivalent LV SV in VFD. An additional finding in VFD-reared females was a reduced arterial pulse pressure in comparison to non-VFD. The reduced pulse pressure was directly correlated with LV cardiac dimension, and arose from numerically lower systolic BP and numerically higher diastolic BP, suggesting the LV of VFD was performing less cardiac “work”. Effects were controlled for age, body mass, crown-rump length and when appropriate, sex. Thus, despite LV hypotrophy, basal SV was maintained in VFD, presumably via a compensatory increase in LV EF. It is conceivable therefore that LV function would be compromised upon exertion due to less contractile reserve, resulting in a “ceiling effect” for LV EF during exertion. Parallel “thrift” adaptations were evident across peripheral and central bodily functions: timid responses in males to the human intruder paradigm, a putatively calorically-sparing behavior, were associated with a relatively reduced LV dimension in contrast to those with a confrontational response. Moreover, in males, timid subjects exhibited reduced SV in comparison to confrontational subjects. SV in males was also directly correlated with relatively reduced neurotrophic status of the corpus callosum white matter – as reflected by cross-sectional area. In addition, in support of a central/peripheral interaction, both SV and LVEDD were inversely correlated with CSF concentrations of the stress neuropeptide CRF. Increases in CRF, a marker of ELS, would be expected to predict cardiac hypotrophy. The marked effect size of the relationship between certain cardiac parameters -- positively to neurotrophic markers and inversely to stress-related biomarkers -- suggests that, although preliminary, consideration of maladaptive thrift alterations may be relevant to certain mood and anxiety disorders. In summary, LV hypotrophy following early life stress may be viewed as consistent with a thrift adaptation with parallel convergent neurobehavioral correlates.

Although prenatal exposure to hypoxia and oxidative stress are deleterious to cardiac developmental programming (Giussani et al., 2012) and prenatal maternal stress adversely affects the offspring's neurobehavioral development (Van den Bergh et al., 2005), the VFD experimental model permits an objective evaluation of the postnatal relationship of ELS to cardiac structure and function, presumably without prenatal confounds. Moreover, since caloric restriction of infants is averted (Coplan et al., 1996), observed effects are likely to stem from the impact of disruption of the maternal repertoire, an emotional effect (Coplan et al., 1995). Gluckman et al. (2009) argue that postnatal epigenetic modifications in DNA methylation and histone modification facilitate “developmental plasticity evolved to match an organism to its environment”. The authors posit that “mismatch between the phenotypic outcome of adaptive plasticity and the current environment” increases the risk of cardiovascular disease, although the same group invoke intrauterine nutritional influences as “perhaps” more relevant (Godfrey et al., 2007). The finding of reduced LV dimension following VFD-rearing would be consistent with a state of energy thrift since LV load is a major determinant of myocardial oxygen consumption (Binak et al., 1967). As postnatal growth of the heart is almost entirely reliant on hypertrophy of individual cardiomyocytes (Porrello et al., 2008), the data suggest an attenuation of normative cardiomyocyte hypertrophy across development. In Coplan et al. (1996), a third control condition was included which was not variable but persistently high foraging conditions (HFD) for mothers. In that group, the uncertainty of foraging (VFD) was removed and mothers worked twice as hard as variable foraging demand conditions. CSF CRF concentrations in offspring were elevated in the VFD-reared group but not the HFD or low foraging demand (LFD) group, arguing that the effects on VFD offspring were psychosocial and not nutritional or effort-related in nature.

The results reveal three measures in which the two sexes were directly compared and that showed significant sex effects: 1) larger overall LV dimension in males; 2) sex as a significant predictor of both LVEDD and LVESD; and, 3) females exhibiting greater % values for LVEF than males. No sex x rearing group effects was noted. Therefore, it should be noted that for the primary variables of the study, sex effects were noted. Because cardiovascular data (HR, BP) were only available in females and neurobehavioral measures only available in males, the current paper is limited by not permitting a more thorough examination of sex effects. Otherwise sex was considered a covariate and controlled for appropriately.

The findings of the present study differ from those of two prior studies. In a rat model of intermittent social isolation, Carlier et al. found stressed rats to have higher systolic blood pressure and higher levels of ornithine decarboxylase activity, a marker for hypertrophy, in the right ventricle (Carlier et al., 1988). Del Duca et al. found hypoxia exposure as an early life stress in rodents to be associated with LV hypertrophy weeks after induction of the stress (Del Duca et al., 2012). Differing results between the present and prior studies are likely related to different animal models utilized and types of stress induced. The possibility is also raised that the findings of the current study represent aberrant structural development due to inconsistent early mothering. Future studies are warranted.

Since arterial pulse pressure was lower in the female VFD-reared group, we assessed total arterial elastance (stroke volume/pulse pressure) and found no significant differences between the groups. Correlation analyses suggest that lower pulse pressure in the VFD female group was related to reduced cardiac dimensions and not to altered aortic properties, the latter having been noted following adverse childhood experiences in humans (Su et al., 2014).

Bonnet females reach sexual maturity (under lab conditions) at approximately 2.5–3.0 years and full body size about 2.5–3 years later (53). Males under similar lab conditions reach sexual maturity at about 4.0–5.0 yrs and full body size about 2–3 years later. Although males and females differed in chronological years, members of each sex in the current study were several years beyond puberty and had reached full body size at the time of testing. Thus, the sexes were thus “developmentally” well-matched.

Limitations include that blood pressure data were available only in females and neurobehavioral correlates were available only in males. However, echocardiographic data were obtained in both male and female monkeys and controlled for sex effects. Thus, although significant findings were separately noted for males and females, these remain non-generalizable to both sexes. Since the ages of ∼8.7 years for males and ∼4.7 years for female were significantly different, an age confound is introduced when examining sex effects. Given that at these ages, all subjects were fully grown, as described above, we contend that this confound would be relatively minimized. Nevertheless for all sex effects examined, age is used as a covariate (see results). Analyses indicated that whereas both sex and weight did significantly predict both LVEDD and LVESD, age did not predict either. The suggestion, therefore, is that sex and weight differences rather than age differences are most salient to cardiac dimension.

Mother's milk is the complete source of nutrition initially, with infant sampling of solids by about month 2 and in increasing amounts thereafter, generally fully weaned by 9–12 months (Kaufman and Rosenblum, 1966). Thus, the VFD exposure period generally occurs during the weaning period, which, since it is an extended process, is expected to have a variable effect on the maternal-infant dyad. The investigators observed no long-lasting effects on macaque offspring when mothers are divided by an “early” versus “late” form of VFD exposure, but a significant impact on maternal cerebrospinal fluid CRF concentrations were evident (Coplan et al., 2006). In human breast milk, there is a moderating role of maternal cortisol on offspring BMI with high breast milk cortisol being associated with high BMI (Hahn-Holbrook et al., 2016). The experimental obstacles to tracking nonhuman primate breast milk cortisol levels through the duration of VFD exposure paradigm without major perturbation of dyadic stress levels would need to be overcome. Also of note was that CSF cortisol concentrations were reduced in the VFD versus LFD subjects and VFD versus HFD such that elevated breast milk cortisol in VFD mothers was unlikely (Coplan et al., 1996).

Controlling for gonadal hormones may provide additional rigor. Additionally, epigenetic analyses would be required to detect differential methylation in response to the VFD form of early life stress. Another limitation is the hemodynamic effects of anesthesia on LV contractility and blood pressure but both groups were given weight-based doses of ketamine. Another major limitation of the current study is that energy expenditure of heart muscle was not directly measured which would test certain aspects of the hypothesis more directly. As invoked above, caloric sparing may occur most obviously upon exertion and not at basal levels. Moreover, it should be noted that the echocardiographic effects documented in the current study are persistent, and minor cardiac alterations may yield caloric thrift only over an extended period of time. An additional limitation, individual caging of social animals-- in this case of adult fully grown males -- is considered a social stressor (House et al., 1988). However, injuries to adult males are prohibitively high in social groups as males compete for social dominance in a harem social structure. Also, these males had been socially housed until they presented a physical danger.

Important questions concerning critical aspects of the VFD model – relevance of age of onset, relationship of maternal nursing versus infant food procurement, how the VFD model alters the bonnet macaque maternal-infant dyad's first year of life -- should be noted but discussion of the detailed aspects of the VFD model would greatly exceed the scope of the current paper. Specific details, however, of the experimental development of the model and how it interacts with the maternal-infant relationship have been reported. The reader is referred to earlier publications detailing the establishment of the VFD model (Coplan et al., 1996, Jackowski et al., 2011). In order to quickly familiarize the reader with some detailed information concerning the first year of the bonnet macaque maternal-infant relationship, a section derived from Kaufman and Rosenblum (1966) is included in the appendix.

As expressed in the introduction, caloric deprivation, likely to be reflected in differential growth patterns, does not appear to be relevant to the current data since previous research has shown that developmental weight gains in VFD-reared infants do not differ from their non-VFD counterparts (Anda et al., 1999).

Another limitation is that data in the current manuscript were measured only at a single time point. Although data derived from a range of developmental stages might be ideal, frequent interventions and assessments of both mothers and their infants would likely add to the early stress levels of dyads under both rearing conditions and thus potentially thorough differential findings of interest. Larger cross-sectional complements of matched subjects would overcome this problem, but such populations, often assessable in rodent studies for example, are not available for nonhuman primate research.

Bonnet macaque subjects give birth to one offspring at a time potentially every mating season or one per year (Kaufman and Rosenblum, 1966) but this is avoided in the laboratory to avoid shared maternal environment effects so it is unlikely but feasible that share maternal environment may have occurred. This was also unlikely in a breeding, social colony of bonnet macaques of almost 400 subjects. Future studies should control for common parity. A number of maternal factors including obesity and age of the mothers can contribute adversely to developmental programming (Armitage et al., 2004). These are not variables we have routinely recorded but warrant attention in future studies.

In conclusion, the findings include decreases in LV systolic dimension and increases in EF in bonnet macaques exposed to ELS. Although SV, perhaps through increased EF, is preserved in VFD-versus non-VFD reared subjects, the finding of reduced pulse pressure in females implies ELS effects not only on structure but also cardiac function. Consistent with a “thrifty phenotype” hypothesis, the putative strategies for caloric sparing within the cardiovascular system are mirrored by similar putative strategies in the affective domain – timid behavioral response and atrophy of the white matter of the corpus callosum. Moreover, LV dimension reductions in females were inversely related to the anorexogenic stress neuropeptide, CRF, a known mediator of ELS (Coplan et al., 1996) and mood and anxiety disorders (Heim and Nemeroff, 2001). Our findings support the hypothesis that ELS, independent of caloric privation, may produce putative epigenetic thrift effects that are similar to direct caloric privation, and that these effects are evident peripherally and correlate with neurobehavioral parameters relevant to anxiety and mood disorders susceptibility.

## Appendix

### Bonnet macaque rearing patterns (Kaufman and Rosenblum, 1966)

Infant macaques are carried on their mother's ventrum for diminishing periods throughout the first year of life, always sleeping on her during that period. Mother's milk is the complete source of nutrition initially, with infant sampling of solids by about month 2 and in increasing amounts thereafter, generally fully weaned by 9–12 months. Mothers continuously carry their infants during daytime for about the first two months with increasing durations of separation most pronounced after about month 5. Mothers may carry or increasingly leave behind their growing infants as they forage or engage in social interactions. Under natural conditions, females, following a 5.5 month gestation may have another infant after a year or may skip a year depending on seasonal factors.”

## References

[bib1] Alastalo H., Räikkönen K., Pesonen A., Osmond C., Barker D., Heinonen K. (2013). Early life stress and blood pressure levels in late adulthood. J. Hum. Hypertens..

[bib2] Albanese A., Hamill G., Jones J., Skuse D., Matthews D., Stanhope R. (1994). Reversibility of physiological growth hormone secretion in children with psychosocial dwarfism. Clin. Endocrinol..

[bib3] Alciati A., Gesuele F., Casazza G., Foschi D. (2013). The relationship between childhood parental loss and metabolic syndrome in obese subjects. Stress Health.

[bib4] Anda R.F., Croft J.B., Felitti V.J., Nordenberg D., Giles W.H., Williamson D.F. (1999). Adverse childhood experiences and smoking during adolescence and adulthood. Jama.

[bib5] Andrews M.W., Rosenblum L.A. (1991). Attachment in monkey infants raised in variable-and low-demand environments. Child. Dev..

[bib6] Armitage J.A., Khan I.Y., Taylor P.D., Nathanielsz P.W., Poston L. (2004). Developmental programming of the metabolic syndrome by maternal nutritional imbalance: how strong is the evidence from experimental models in mammals?. J. physiology.

[bib7] Bercovich E., Keinan-Boker L., Shasha S.M. (2014). Long-term health effects in adults born during the Holocaust. Israel Med. Assoc. J. IMAJ.

[bib8] Binak K., Harmanci N., Sirmaci N., Ataman N., Ogan H. (1967). Oxygen extraction rate of the myocardium at rest and on exercise in various conditions. Br. heart J..

[bib9] Carlier P.G., Crine A.F., Yerna N.M., Rorive G.L. (1988). Cardiovascular structural changes induced by isolation-stress hypertension in the rat. J. Hypertens..

[bib10] Coplan J.D., Rosenblum L.A., Gorman J.M. (Dec 1995). Primate models of anxiety: longitudinal perspectives. Psychiatric Clin. N. Am..

[bib11] Coplan J.D., Andrews M.W., Rosenblum L.A., Owens M.J., Friedman S., Gorman J.M. (1996). Persistent elevations of cerebrospinal fluid concentrations of corticotropin-releasing factor in adult nonhuman primates exposed to early-life stressors: implications for the pathophysiology of mood and anxiety disorders. Proc. Natl. Acad. Sci..

[bib12] Coplan J.D., Smith E., Trost R.C., Scharf B.A., Altemus M., Bjornson L. (2000). Growth hormone response to clonidine in adversely reared young adult primates: relationship to serial cerebrospinal fluid corticotropin-releasing factor concentrations. Psychiatry Res..

[bib13] Coplan J.D., Altemus M., Mathew S.J., Smith E.L., Sharf B., Coplan P.M. (2005). Synchronized maternal-infant elevations of primate CSF CRF concentrations in response to variable foraging demand. CNS spectrums.

[bib14] Coplan J.D., Smith E.L., Altemus M., Mathew S.J., Perera T., Kral J.G. (2006). Maternal–infant response to variable foraging demand in nonhuman primates. Ann. N. Y. Acad. Sci..

[bib15] Coplan J.D., Abdallah C.G., Kaufman J., Gelernter J., Smith E.L., Perera T.D. (2011). Early-life stress, corticotropin-releasing factor, and serotonin transporter gene: a pilot study. Psychoneuroendocrinology.

[bib16] Coplan J.D., Fulton S.L., Reiner W., Jackowski A., Panthangi V., Perera T.D. (2014). Elevated cerebrospinal fluid 5-hydroxyindoleacetic acid in macaques following early life stress and inverse association with hippocampal volume: preliminary implications for serotonin-related function in mood and anxiety disorders. Front. Behav. Neurosci..

[bib17] Del Duca D., Tadevosyan A., Karbassi F., Akhavein F., Vaniotis G., Rodaros D. (2012). Hypoxia in early life is associated with lasting changes in left ventricular structure and function at maturity in the rat. Int. J. Cardiol..

[bib18] Dong M., Giles W.H., Felitti V.J., Dube S.R., Williams J.E., Chapman D.P. (2004). Insights into causal pathways for ischemic heart disease adverse childhood experiences study. Circulation.

[bib19] Felitti V.J., Anda R.F., Nordenberg D., Williamson D.F., Spitz A.M., Edwards V. (1998). Relationship of childhood abuse and household dysfunction to many of the leading causes of death in adults: the Adverse Childhood Experiences (ACE) Study. Am. J. Prev. Med..

[bib20] Garcia-Rizo C., Fernandez-Egea E., Bernardo M., Kirkpatrick B. (2015). The thrifty psychiatric phenotype. Acta Psychiatr. Scand..

[bib21] Giussani D.A., Camm E.J., Niu Y., Richter H.G., Blanco C.E., Gottschalk R. (2012). Developmental programming of cardiovascular dysfunction by prenatal hypoxia and oxidative stress. PloS one.

[bib22] Gluckman P.D., Hanson M.A., Buklijas T., Low F.M., Beedle A.S. (2009). Epigenetic mechanisms that underpin metabolic and cardiovascular diseases. Nat. Rev. Endocrinol..

[bib23] Godfrey K.M., Lillycrop K.A., Burdge G.C., Gluckman P.D., Hanson M.A. (2007). Epigenetic mechanisms and the mismatch concept of the developmental origins of health and disease. Pediatr. Res..

[bib24] Hahn-Holbrook J., Le T.B., Chung A., Davis E.P., Glynn L.M. (2016). Cortisol in human milk predicts child BMI. Obesity.

[bib25] Haider B., Yeh C., Thomas G., Oldewurtel H., Lyons M., Regan T. (1977). Altered myocardial function and collagen in diabetic rhesus monkeys on atherogenic diet. Trans. Assoc. Am. Physicians.

[bib26] Hales C.N., Barker D.J. (1992). Type 2 (non-insulin-dependent) diabetes mellitus: the thrifty phenotype hypothesis. Diabetologia.

[bib27] Heim C., Nemeroff C.B. (2001). The role of childhood trauma in the neurobiology of mood and anxiety disorders: preclinical and clinical studies. Biol. psychiatry.

[bib28] House J.S., Landis K.R., Umberson D. (1988). Social relationships and health. Science.

[bib29] Jackowski A., Perera T.D., Abdallah C.G., Garrido G., Tang C.Y., Martinez J. (2011). Early-life stress, corpus callosum development, hippocampal volumetrics, and anxious behavior in male nonhuman primates. Psychiatry Res. Neuroimaging.

[bib30] Kaufman I.C., Rosenblum L.A. (1966). A behavioral taxonomy forMacaca nemestrina andMacaca radiata: based on longitudinal observation of family groups in the laboratory. Primates.

[bib31] Kaufman D., Smith E.L., Gohil B.C., Banerji M., Coplan J.D., Kral J.G. (2005). Early appearance of the metabolic syndrome in socially reared bonnet macaques. J. Clin. Endocrinol. Metabolism.

[bib32] Kaufman D., Banerji M.A., Shorman I., Smith E.L., Coplan J.D., Rosenblum L.A. (2007). Early-life stress and the development of obesity and insulin resistance in juvenile bonnet macaques. Diabetes.

[bib33] Kinnally E.L., Feinberg C., Kim D., Ferguson K., Leibel R., Coplan J.D. (2011). DNA methylation as a risk factor in the effects of early life stress. Brain, Behav. Immun..

[bib34] Lang R.M., Bierig M., Devereux R.B., Flachskampf F.A., Foster E., Pellikka P.A. (2005). Recommendations for chamber quantification: a report from the American society of Echocardiography's guidelines and standards committee and the chamber quantification writing group, developed in conjunction with the European association of echocardiography, a branch of the European society of cardiology. J. Am. Soc. Echocardiogr..

[bib35] Lazar J.M., Qureshi G., Qureshi M.R., Smith E., Scharf B., Rosenblum L.A. (2008). Left ventricular systolic and diastolic function in healthy adult bonnet macaques (Macaca radiata). New echocardiographic indices in Old World monkeys. Cardiology.

[bib36] Lazar J., Qureshi G., Kamran H., Rosenblum L.A., Kral J.G., Salciccioli L. (2009). Characterization of arterial wave reflection in healthy bonnet macaques: feasibility of applanation tonometry. BioMed Res. Int..

[bib37] Loria A.S., Pollock D.M., Pollock J.S. (2010). Early life stress sensitizes rats to angiotensin II–induced hypertension and vascular inflammation in adult life. Hypertension.

[bib38] Loria A.S., Ho D.H., Pollock J.S. (2014). A mechanistic look at the effects of adversity early in life on cardiovascular disease risk during adulthood. Acta physiol..

[bib39] Molteni R., Wu A., Vaynman S., Ying Z., Barnard R., Gomez-Pinilla F. (2004). Exercise reverses the harmful effects of consumption of a high-fat diet on synaptic and behavioral plasticity associated to the action of brain-derived neurotrophic factor. Neuroscience.

[bib40] Money J. (1977). The syndrome of abuse dwarfism (psychosocial dwarfism or reversible hyposomatotropism): behavioral data and case report. Am. J. Dis. Child..

[bib41] Nuyt A. (2008). Mechanisms underlying developmental programming of elevated blood pressure and vascular dysfunction: evidence from human studies and experimental animal models. Clin. Sci..

[bib42] Ockenburg S., Tak L., Bakker S., Gans R., Jonge P., Rosmalen J. (2015). Effects of adverse life events on heart rate variability, cortisol, and C-reactive protein. Acta Psychiatr. Scand..

[bib43] Pelleymounter M.A., Joppa M., Carmouche M., Cullen M.J., Brown B., Murphy B. (2000). Role of corticotropin-releasing factor (CRF) receptors in the anorexic syndrome induced by CRF. J. Pharmacol. Exp. Ther..

[bib44] Perera T.D., Lu D., Thirumangalakudi L., Smith E.L., Yaretskiy A., Rosenblum L.A. (2011). Correlations between hippocampal neurogenesis and metabolic indices in adult nonhuman primates. Neural plast..

[bib45] Porrello E.R., Widdop R.E., Delbridge L. (2008). Early origins of cardiac hypertrophy: does cardiomyocyte attrition programme for pathological ‘Catch-up’growth of the heart?. Clin. Exp. Pharmacol. Physiology.

[bib46] Reinhart J.B., Drash A.L. (1969). Psychosocial dwarfism: environmentally induced recovery. Psychosom. Med..

[bib47] Rosenblum L.A., Paully G.S. (1984). The effects of varying environmental demands on maternal and infant behavior. Child. Dev..

[bib48] Rosenblum L.A., Forger C., Noland S., Trost R.C., Coplan J.D. (2001). Response of adolescent bonnet macaques to an acute fear stimulus as a function of early rearing conditions. Dev. Psychobiol..

[bib49] Sánchez M.M., Hearn E.F., Do D., Rilling J.K., Herndon J.G. (1998). Differential rearing affects corpus callosum size and cognitive function of rhesus monkeys. Brain Res..

[bib50] Sawilowsky S., Sawilowsky J., Grissom R.J. (2011). Effect Size. International Encyclopedia of Statistical Science.

[bib51] Steptoe A., Kivimäki M. (2012). Stress and cardiovascular disease. Nat. Rev. Cardiol..

[bib52] Steptoe A., Kivimäki M. (2013). Stress and cardiovascular disease: an update on current knowledge. Annu. Rev. public health.

[bib53] Su S., Wang X., Kapuku G.K., Treiber F.A., Pollock D.M., Harshfield G.A. (2014). Adverse childhood experiences are associated with detrimental hemodynamics and elevated circulating endothelin-1 in adolescents and young adults. Hypertension.

[bib54] Teicher M.H., Dumont N.L., Ito Y., Vaituzis C., Giedd J.N., Andersen S.L. (2004). Childhood neglect is associated with reduced corpus callosum area. Biol. psychiatry.

[bib55] Vaitkevicius P.V., Lane M., Spurgeon H., Ingram D.K., Roth G.S., Egan J.J. (2001). A cross-link breaker has sustained effects on arterial and ventricular properties in older rhesus monkeys. Proc. Natl. Acad. Sci..

[bib56] Van den Bergh B.R., Mulder E.J., Mennes M., Glover V. (2005). Antenatal maternal anxiety and stress and the neurobehavioural development of the fetus and child: links and possible mechanisms. A review. Neurosci. Biobehav. Rev..

[bib57] Weder N., Zhang H., Jensen K., Yang B.Z., Simen A., Jackowski A. (2014). Child abuse, depression, and methylation in genes involved with stress, neural plasticity, and brain circuitry. J. Am. Acad. Child Adolesc. Psychiatry.

